# *Agrobacterium* spp. nosocomial outbreak assessment using rapid MALDI-TOF MS based typing, confirmed by whole genome sequencing

**DOI:** 10.1186/s13756-019-0619-y

**Published:** 2019-11-04

**Authors:** Carlo Casanova, Elia Lo Priore, Adrian Egli, Helena M. B. Seth-Smith, Lorenz Räber, Daniel Ott, Valentin Pflüger, Sara Droz, Jonas Marschall, Rami Sommerstein

**Affiliations:** 1 Institute for Infectious Diseases, University of Bern, Friedbühlstrasse 51, 3001 Bern, Switzerland; 2 Department of Infectious Diseases, Bern University Hospital, University of Bern, Freiburgstrasse, 3001 Bern, Switzerland; 3 Division of Clinical Microbiology, University Hospital Basel, Basel, Switzerland; 4 Applied Microbiology Research, Department of Biomedicine, University of Basel, Basel, Switzerland; 5 Department of Cardiology, Bern University Hospital, University of Bern, Bern, Switzerland; 6 Department of Radiology, Bern University Hospital, University of Bern, Bern, Switzerland; 7 Mabritec AG, Riehen, Switzerland

**Keywords:** *Agrobacterium*, *Rhizobium*, Nosocomial outbreak, MALDI-TOF MS, Whole genome sequencing

## Abstract

**Background:**

A number of episodes of nosocomial *Agrobacterium* spp*.* bacteremia (two cases per year) were observed at Bern University Hospital, Switzerland, from 2015 to 2017. This triggered an outbreak investigation.

**Methods:**

Cases of *Agrobacterium* spp*.* bacteremias that occurred between August 2011 and February 2017 were investigated employing line lists, environmental sampling, rapid protein- (MALDI-TOF MS), and genome-based typing (pulsed field gel electrophoresis and whole genome sequencing) of the clinical isolates.

**Results:**

We describe a total of eight bacteremia episodes due to *A. radiobacter* (*n* = 2), *Agrobacterium* genomovar G3 (*n* = 5) and *A. pusense* (*n* = 1). Two tight clusters were observed by WGS typing, representing the two *A. radiobacter* isolates (cluster I, isolated in 2015) and four of the *Agrobacterium* genomovar G3 isolates (cluster II, isolated in 2016 and 2017), suggesting two different point sources. The epidemiological investigations revealed two computer tomography (CT) rooms as common patient locations, which correlated with the two outbreak clusters. MALDI-TOF MS permitted faster evaluation of strain relatedness than DNA-based methods. High resolution WGS-based typing confirmed the MALDI-TOF MS clustering.

**Conclusions:**

We report clinical and epidemiological characteristics of two outbreak clusters with *Agrobacterium.* spp. bacteremia likely acquired during CT contrast medium injection and highlight the use of MALDI-TOF MS as a rapid tool to assess relatedness of rare gram-negative pathogens in an outbreak investigation.

## Background

Bacteria of the genus *Agrobacterium* are environmental gram-negative rods, present in soil and plants. They are rare opportunistic human pathogens, with the majority of infections reported in immunocompromised hosts, such as oncology patients, the elderly, and neonates [1–3]. Central venous catheter (CVC)-related bloodstream infection (BSI) is the most common clinical presentation and is occasionally preceded by exposure to soil [4, 5]. Other clinical manifestations include spondylodiscitis, endophthalmitis, pneumonia, peritonitis, urinary tract infection and endocarditis [6–11]. Furthermore, an *Agrobacterium* strain has been recognized as a cause of pseudobacteremia following contamination of blood cultures in the microbiology laboratory [12]. Although nosocomial cases have been described before, a common source has never been reported [8, 11]. Recently, *A. radiobacter* strains were reported in a three-species outbreak in Brazil associated with total parenteral nutrition [13].

The taxonomy and nomenclature of the genus *Agrobacterium* has been subject of a long debate [14, 15] but has now come to a consensus [16–19]. To date, at least 20 genomospecies are recognized [20] whereof many are yet to receive valid latin binomial names. Notably, a number of previous clinical reports on strains of “*Rhizobium radiobacter*” and “*Agrobacterium tumefaciens*” point at the same species, now officially termed *Agrobacterium radiobacter* (= genomovar G4 of the “*A. tumefaciens* species complex”) [19].

A variety of molecular techniques are available to determine relatedness of bacterial isolates in outbreak situations [21]. Pulsed-field gel electrophoresis (PFGE) has been considered the “gold standard” typing method for many bacterial species; however, the method is not easily transferable between laboratories and requires a high level of standardization. Next-generation sequencing technologies are currently becoming more widely available for medical microbiology laboratories and typing by analysis of whole genome sequencing (WGS) data is the method offering the highest discriminatory power [22]. The introduction of MALDI-TOF MS has revolutionized clinical bacteriology and is used in many laboratories for rapid identification of most clinically relevant bacterial species at very low cost. Beyond species identification, the utility of MALDI-TOF MS-based typing for outbreak investigation has been demonstrated for several pathogens, including *Acinetobacter baumannii, Enterobacter cloacae* and *Escherichia coli* [23–28]. For *Enterococcus faecium* and *Staphylococcus aureus*, however, it is more controversially discussed whether the discriminatory power of MALDI-TOF MS is sufficient for strain typing [24, 29, 30], indicating that the applicability of this technique may depend on the bacterial species. Here, we investigated a series of nosocomial *Agrobacterium* spp*.* bacteremia episodes observed at Bern University Hospital, Switzerland, and used MALDI-TOF MS-based typing as first-line method to determine relatedness of the isolates, which was subsequently confirmed by high-resolution WGS-based typing.

### The event (index cases)

**Index case A** was a 72-year-old male admitted on November 26, 2015 for an elective transcatheter aortic valve implantation. A computed tomography (CT)-angiography was performed on the admission day in CT room 1. The next day, the patient underwent diagnostic cardiac catheterization in the catheter laboratory A, followed by transcatheter aortic valve implantation in the hybrid procedure room. Three days later he developed fever with rigors, an elevated C-reactive protein (CRP) of 147 mg/l, and a leucocyte count of 15.0 G/L. At time of symptom onset, the left internal jugular CVC and a percutaneous sheath introducer, both inserted upon hospital admission, had already been removed. No alternative focus of infection was found. Both peripherally drawn blood cultures revealed growth of *A. radiobacter*. The tip culture of the removed CVC showed no growth. Antimicrobial treatment included empirical intravenous amoxicillin-clavulanate, cefepime and later ceftriaxone for a total duration of 10 days. The clinical course was favorable (Additional file 1: Figure S1).

**Index case B** was an 83-year-old female admitted on December 1, 2015 with acute congestive heart failure in the setting of severe aortic stenosis. She underwent diagnostic cardiac catheterization in catheter laboratory A on the same day. CT-angiography was performed 2 days later in CT room 1. On day three after catheterization she developed fevers with chills accompanied by an elevated CRP of 19 mg/l and a leucocyte count of 12.5 G/L. The only vascular access present was a peripheral venous catheter on the left forearm inserted upon hospital admission. No other potential focus of infection was detected. Both blood cultures drawn were positive for *A. radiobacter*. The peripheral catheter tip was not cultured. The patient was empirically treated with intravenous amoxicillin-clavulanate, followed by meropenem plus ciprofloxacin for a total duration of 14 days. The clinical course was favorable (Additional file 1: Figure S1).

These two index cases triggered the outbreak investigation.

## Methods

### Epidemiological and clinical investigation

A case was defined as a patient with an *Agrobacterium* or *Rhizobium* sp*.* isolate from at least one blood culture during the hospital stay at Bern University Hospital, a 950-bed tertiary care center. Potential cases were reported by the infectious diseases consultation service and/or identified via a structured query of the microbiology laboratory’s database with the search criteria [“*Agrobacterium”* OR “*Rhizobium”*] AND “blood culture” in the time period from 01 January 2011 to 31 December 2017. We then collected data on clinical and epidemiological baseline characteristics of the cases as well as treatment and outcome data.

We generated a line list in order to identify possible common sources. This was followed by on-site audits of identified common locations and environmental swabs of suspected sources. Environmental sampling was performed with premoistened swabs, cultured on MacConkey agar and CHROMagar™ Orientation (Becton Dickinson, Sparks, USA) **(**Additional file 1: Table S1).

### Identification of blood culture isolates

Blood cultures were taken according to clinical routine and only if clinically indicated. Inoculated BacT/ALERT® FA plus (aerobic) and FN plus (anaerobic) blood culture bottles were incubated in the BacT/ALERT® 3D (before November 2016) or the VIRTUO® System (bioMérieux, Inc. Marcy l’Etoile, France). Initial identification was performed on blood culture isolates cultured at 35 °C in CO_2_ enriched atmosphere by MALDI-TOF MS (Bruker Biotyper; Bruker Daltonics, Bremen, Germany) using the MBT 6903 MSP Library, and for selected isolates with 16S rRNA gene sequencing using the MicroSeq®500 16S rDNA PCR and Sequencing Kits (Applied Biosystems, Foster City, CA).

### MALDI-TOF MS- based typing

MALDI-TOF MS-based typing was performed as described previously, with minor modifications [23]. In brief, fresh sub-cultured isolates were used and, after full protein extraction with formic acid, four spectra were generated for each isolate with a Microflex™ MALDI-TOF (Bruker Daltonics). Using the flexAnalysis™ software version 3.4 (Bruker Daltonics) spectra were visually examined in the “overlaid” and “list view” mode for differentiating peaks among the eight isolates. Due to slow growth of the subcultures the incubation time was extended from 24 h to 48 h to yield more pronounced *m/z* peaks. A peak list was generated with peaks with a signal intensity of > 1000 arbitrary units; peaks with a signal-to-noise ratio < 10 were only included in the analysis if they were visually clearly distinguishable from background level (Additional file 1: Figure S2). From the resulting binary peak list a distance matrix (dissimilarity structure) was computed (function *dist*) and a hierarchical cluster (function *hclust*) was generated in R [31]. Principal component analysis (PCA) was performed with MALDI Biotyper Compass Explorer 4.1 using standard settings.

### Genome-based typing

Pulsed-field gel electrophoresis (PFGE): PFGE was performed with chromosomal DNA digested with *Pme* I as previously described [32].

WGS: All isolates were sequenced on an Illumina MiSeq, 2x300bp following Nextera XT library preparation. Data analysis was performed in CLC genomics Workbench v9.5.3. All samples were mapped against a de novo assembly of isolate F, showing that mean sequencing coverage of all isolates was over 58x. A further within-cluster comparison was based on the mapping against the assembly of the genome of isolate B. Variants were called at 10x minimum coverage, 10 minimum count and 70% minimum frequency. Single nucleotide polymorphism (SNP) tree generation used a neighbor-joining method: minimum coverage 10%, minimum z-score 1.96, multi-nucleotide variants included. Digital DNA:DNA hybridization [33] was performed against all known *Agrobacterium* genomospecies [18] and confirmed using average nucleotide identity (ANI; http://enve-omics.ce.gatech.edu/ani/). Mapping was also performed against plasmids pTi-SAKURA (accession number NC_002147.1) and pRi1724 (NC_002575.1), showing low (< 20%) fraction of plasmids covered at low (<10x) coverage. The datasets supporting the conclusions of this article are available in the European Nucleotide Archive repository, under Project number PRJEB34002 [https://www.ebi.ac.uk/ena/data/view/PRJEB34002] (Additional file 1: Table S2).

## Results

### Epidemiological and clinical investigation for the time period 2011–2017

In a two-week period between November 29 and December 7, 2015, we isolated *A. radiobacter* from blood cultures of two patients (Isolates A and B, =cluster I) admitted to the cardiology department. A close relation between the two isolates was suspected after rapid visual examination of their MALDI-TOF MS profiles (data not shown).

In October 2016 two additional patients presented with *Agrobacterium* sp*.* bloodstream infections within a two-week period (Isolates C and D; = cluster II) and two more cases occurred in January and February 2017 (Isolates E and F; = cluster II). The isolates were identified with MALDI-TOF MS as *Agrobacterium sp*. with *A. radiobacter* as best match. These additional cases led to an extended epidemiological and clinical investigation encompassing all cases between 2011 and 2017 at Bern University Hospital. Two further cases (Isolates G and H) were identified, resulting in a total of eight cases.

The clinical characteristics of the eight patients are provided in Tables 1 and 2. The median age was 53 years (range 18–83 years), the majority were male (63%). Three patients were admitted to medical wards, three to a surgical ward, one was treated in the emergency room and one in the radiology outpatient clinic.

**Table 1:** Epidemiological data of eight *Agrobacterium* cases

Patient	Age	Sex	Clinical service	Date of hospital admission	Cluster	CT-scan Room n°	Date of CT-angiogram	Date of first positive BC	Δt CT-positive BC	Isolate	Other invasive procedures
1	47	M	Medicine	23.08.2011	NA	CT1-R	27.07.11	23.08.11	26d 23 h 44 m	G	none
2	38	F	Oncology	07.04.2013	NA	CT2-ER	12.04.13	02.05.13	19d 17 h 39 m	H	Repeated self-injection of sedatives
3	72	M	Surgery	26.11.2015	1	CT1-R	26.11.15	29.11.15	3d 2 h 34 m	A	TAVI, CA
4	83	F	Surgery	01.12.2015	1	CT1-R	03.12.15	07.12.15	3d 16 h 6 m	B	CA
5	18	M	Medicine	01.10.2016	2	CT2-ER	01.10.16	01.10.16	0d 1 h 4 m	C	none
6	58	M	Emergency Room	12.10.2016	2	CT2-ER	12.10.16	12.10.16	0d 0 h 40 m	D	none
7	81	M	Surgery	10.01.2017	2	CT2-ER	21.01.17	23.01.17	2d 11h22m	E	Coronary bypass-surgery
8	19	F	Radiology	24.02.2017	2	CT2-ER	24.02.17	24.02.17	0d 1 h 1 m	F	none

*Abbreviations: BC* Blood culture, *CA* Coronary angiography, *CT-R* Computed Tomography-scan radiology (=CT Room 1), *CT-ER* Computed Tomography-scan emergency room, *TAVI* Transcatheter aortic valve replacement, *Delta Time* (Δt), *h* hour(s), *m* minute(s), *d*: day(s)

**Table 2:** Clinical data of eight *Agrobacterium* cases

Isolate	Main diagnosis at admission	*Rhizobium*- related Diagnosis^a^	Clinical Features at time of BC sampling	N° positive BC/ BC drawn before treatment	Polymicrobial BSI	IVDs	IVD culture	Antibiotic treatment (days)	Treatment duration (days)	Outcome
A	Elective surgery	CLABSI	Fever, chills	2/2	no	CVC	No growth	Amoxicillin-clavulanic acid (2), Cefepime (3), ceftriaxone (5)	10, CVC removed on day 8	cured
B	Semi-elective surgery	BSI	Fever	2/2	no	PVC	Not performed	Amoxicillin-clavulanic acid (2), Meropenem + Ciprofloxacin (12)	14, removal date of PVC unknown	cured
C	Lung injury from smoke inhalation	Transient bacteraemia	Fever	1/2	no	PVC	Not performed	Amoxicillin-clavulanic acid (3), Ceftriaxone (1)	4	cured
D	Community-acquired pneumonia	Transient bacteraemia	asymptomatic	1/2	no	PVC	Not performed	Amoxicillin-clavulanic acid (7, Doxycyclin (7)	7	cured
E	Acute-myocardial infarction	CRBSI	Fever, chills	2/2	no	PVC	*Agrobacterium* sp.	Piperacillin-tazobactam (2), Ceftraixone (7)	9, PVC removal day 4	cured
F	Polyserositis related to Undifferentiated Connective Tissue Disease	Transient bacteraemia	asymptomatic	1/2	no	PVC	Not performed	Cefepime (3)	3	cured
G	Atypical mycobacterial pulmonary infection	CLABSI	Fever	1/2	Yes; *Ochrobactrum anthrophi*, *Alcaligenes sp.*	Port	Not performed	Imipenem+cilastin	14, Port removal on day 9	cured
H	Self-induced bleeding	CLABSI	Fever	2/3	Yes, *Sphingomonas paucimobilis*, Viridans group Streptococci	CVC	*Sphingomonas paucimobilis*, Viridans group Streptococci	Amoxicillin-clavulanic acid	12, CVC removed on day 6	cured

*Abbreviations*: *BSI* Bloodstream infection, *CLABSI* Central line associated bloodstream infection, *CRBSI* Catheter-related bloodstream infection, *CVC* Central venous catheter, *D* Days, *IVD* Intravascular device, *PVC* Peripheral venous catheter

^a^Each *Agrobacterium*-related case was classified as follows: For central line associated BSI the Centers for Disease Control and Prevention (CDC) / National Healthcare Safety Network (NHSN) definition [34] was used. A catheter-related BSI was defined according to the respective Infectious Disease Society of America guideline [35]. A transient bacteremia was defined as a positive blood culture in an asymptomatic patient

All patients underwent a CT-scan with contrast media injection before developing bacteremia. The time interval between diagnostic radiology and positive blood culture varied with each cluster. For cluster I (Isolates A and B) the median time-to-bacteremia was 81.3 h (range: 74.6–88.1 h); for cluster II (Isolates C, D, E and F) 1.0 h (range: 40 min - 59.4 h); and for cases G and H 26 and 19 days, respectively. Two of eight case patients (G and H) presented with a polymicrobial bloodstream infection. Six patients (75%) presented with fever, the remaining two were afebrile. Three patients (37.5%) presented with a central line-associated bloodstream infection (CLABSI), three (37.5%) with transient bacteremia, one (12.5%) with bloodstream infection and one (12.5%) a catheter-related bloodstream infection. All patients were initially treated with intravenous antibiotics with variable duration (see Table 2 for details) and all patients were cured at the end of treatment without further infection-related complications. In all patients who had an intravascular device at the time of bacteremia, the device was removed.

By means of a line list we identified a commonality for all cases in the form of the intravenous contrast medium injector in two Computer Tomography (CT) rooms (CT Exprés™, Debiotech S. A, Lausanne, Switzerland). For cases A and B we could not exclude a possible common source in the contrast media injector used in the same cardiac catheter laboratory (ACIST CVi™, ACIST Medical System, Eden Prairie, MN, U.S.A.), despite negative microbiological testing (Additional file 1: Figure S1). Beside the epidemiological links, a structural infection risk was identified in the semi-open design of this device (Additional file 1: Figures. S3 and S4). Device contamination could also have been facilitated by construction works adjacent to the cardiac catheter laboratory. Apart from A and B no other patient underwent an invasive cardiology procedure; therefore, we excluded this device as possible common source for all cases. Environmental screening in both, CT scan room and cardiac catheter laboratory, was performed on the contrast media injector pump (including fixed and changeable parts) and contrast media solution. Environmental testing was also performed on plants present in the corridor of the radiology department. **(**Additional file 1: Table S1). None of the cultures revealed growth of *Agrobacterium* spp.

### MALDI-TOF MS-, PFGE- and WGS-based typing

**Fig. 1:**
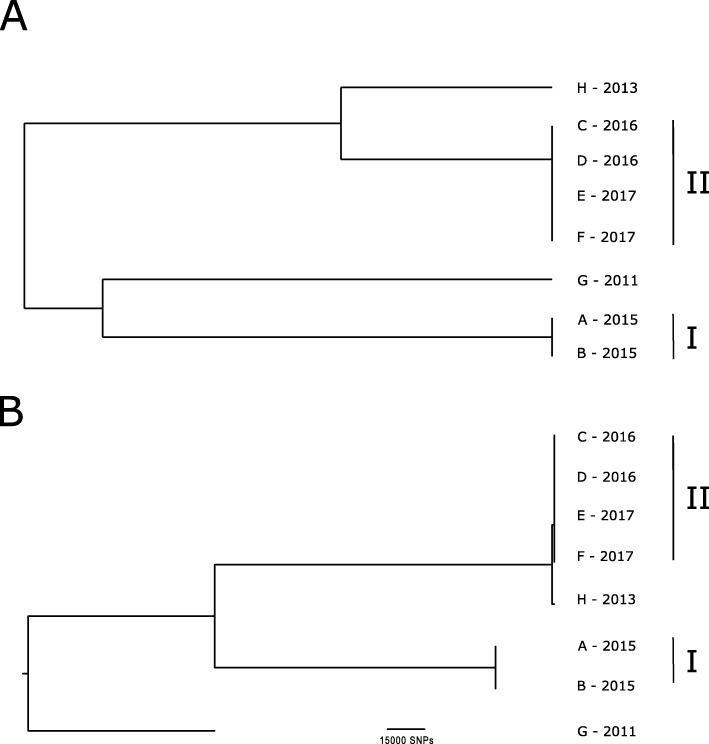
**a** Hierarchical cluster based on observed differences in the MALDI-TOF peak profile of cultures grown for 48 h. Indicated are the year of isolation for isolates A-H and the two outbreak clusters I and II. **b** SNP variant phylogenetic tree of sequenced isolates. The genome of isolate F was used as reference for the phylogeny. Scale bar indicates numbers of SNP differences between the isolates. Isolates H, A/B and G map to only 89, 76 and 77% of the reference genome, respectively

All strains were typed by MALDI-TOF MS, PFGE and WGS. MALDI-TOF MS-typing based on visual examination of the peak profiles and PCA confirmed two temporally related clusters and two outliers (Fig. 1a and Additional file 1: Figure S5): Cluster I (isolates A, B) occurred between November and December 2015 and cluster II (isolates C, D, E, F) between October 2016 and February 2017. According to the MALDI-TOF MS peak list the isolates within these clusters were identical, while a difference of eight *m/z* peaks was recorded to isolate H (Additional file 1: Table S3). Isolate G was unrelated, both chronologically and in terms of MALDI-TOF MS-typing results.

The dendrogram based on PFGE analysis was highly similar to the MALDI-TOF MS dendrogram in clearly delineating the two outbreak clusters I and II and the two separate isolates G and H (Additional file 1: Figure S6).

The WGS phylogenetic results further confirmed the clonality of the two clusters. The genomes of A and B differed by a single nucleotide polymorphism (SNP) (Fig. 1b). Digital DNA:DNA hybridization indicated that this cluster was most closely related to the *A. radiobacter* (= *Agrobacterium* genomovar G4) type strain LMG140^T^ (Additional file 1: Table S4). Cluster II consisted of isolates C, D, E and F, which all contained identical genomes, whereas isolate H shared only 89% of its genome with these four isolates, with the shared portion differing by over 2500 SNPs from the cluster (Fig. 1b), thereby validating the MALDI-TOF MS findings. Larger variable regions differing between these genomes include plasmids, phages, and integrative and conjugative elements / composite transposons. Digital DNA:DNA hybridization indicated that the strains in Cluster II, and isolate H, were most closely related to *Agrobacterium* genomovar G3.

Isolate G, finally, was clearly separated from all other isolates by all typing methods. According to the WGS results it could be assigned to the species *A. pusense* (= *Agrobacterium* genomovar G2). None of the isolates were found to carry pTi or pRi plasmids.

## Discussion

We report an outbreak investigation of eight cases of nosocomial *Agrobacterium* spp. bacteremia at a single tertiary referral center between 2011 and 2017*.* Environmental bacteria growing in blood cultures are usually regarded as contaminants. Members of the genus *Agrobacterium* are, however, recognized as opportunistic human pathogens, particularly in intravascularly catheterized immunocompromised patients [5]. As their isolation from clinical specimen occurs relatively infrequently, temporal clustering of cases in our hospital triggered an outbreak investigation. Despite the fact that no *Agrobacterium* spp. were detected in the contrast medium itself, the epidemiological investigation identified the CT contrast application as the most probable common source of the outbreak. One conceivable transmission pathway is that the pathogen was transferred from the hands of staff to the device and then to the patient. However, the short times to the detection argue for a relatively high inoculum. This would speak for a previous replication in a medium. Since no such medium was found, we must admit that the final transmission mechanism remains unclear. We enforced hand hygiene precautions when handling the CT contrast medium injections and no further cases have since been detected at our institution.

A limitation of this study was the inability to microbiologically confirm either an environmental or a device contamination. Further, no prospective assessment of blood cultures of all patients undergoing CT injection was performed. This could have been reason for bias – possibly, many more patients were affected but never recognized.

For cluster I the cardiac catheterization laboratory remained a suspected common source because of the semi-open design of the contrast medium injector that was found to pose an infectious risk, similar to the risk previously highlighted in other semi-open devices (e.g., syringes) [36]. The clinical outcome was favorable for all patients, as highlighted in other studies [8], irrespective of the use of different treatment regimens which followed the specific antibiogram.

Most isolates described in previous reports on human *Agrobacterium* infections were identified with conventional methods which do not allow differentiation between the various genomovars. In a large study using multilocus sequence-based analysis, the majority of clinical isolates were found to belong to *A. pusense* (genomovar G2) which, consequently, was considered as human-associated genomovar [37].

In our case series only one of the eight isolates was assigned to *A. pusense* (Isolate G), whereas the outbreak clusters I and II consisted of *A. radiobacter* and genomovar G3 strains, respectively. The commercial MALDI-TOF MS database used for the identification of the clinical isolates contains 14 entries for *A. radiobacter*, one for *A. rubi* (in the database deposited as *“Rhizobium radiobacter”* and *“Rhizobium rubi”*) and one for *R. tropici*. Using the Biotyper software, genomovar G2 and G4 isolates were identified with high confidence levels as “*R. radiobacter”* (score > 2.2), while genomovar G3 isolates were identified merely to the genus level (score > 1.7 to < 2.0 for “*R. radiobacter”*).

Using MALDI-TOF MS-typing via visual examination of the peak profiles, the three genomovars clearly fell into three clusters (Fig. 1a), indicating that an identification of individual genomovars based on MS spectra could be achieved using a well-characterized reference database. Comparison of the spectra with a database generated on the base of putatively ascribed ribosomal protein masses extracted from genomic data [38] accordingly allowed a genomovar assignment for all eight isolates (Additional file 1: Table S5). An advanced routine identification method for *Agrobacterium* isolates would in turn lead to a better understanding of the role and prevalence of different *Agrobacterium* genomovars in human infections.

While WGS is currently replacing PFGE as the “gold standard” for molecular epidemiological typing, a growing number of reports demonstrate the utility of MALDI-TOF MS as a rapid, relatively inexpensive method for outbreak investigation. One concern about this method is the current lack of interpretation guidelines for strain relatedness [39] and its lower resolution due to technical limitations. As it is mostly based on the proteome phenotype, which in turn is influenced by horizontal gene transfer of accessory or even ribosomal genes, it does not necessarily reflect evolutionary relatedness. The isolates in our analysis belong to a genus with a high degree of diversity, including in its accessory gene repertoire [40]. The individual genomovars were thus clearly separated by more than 20 peak differences. Furthermore, discrimination of strains within a genomovar was achieved. Isolate H was isolated more than 3 years before the isolates in cluster II but, interestingly, the patient with this isolate underwent contrast medium injection in the same CT room. The isolate could clearly be distinguished by a difference of eight MALDI-TOF MS peaks from the other genomovar G3 isolates, which corresponded to a divergence of 2671 SNPs and 11% of the genome in WGS-based typing.

In this report we highlight the use of MALDI-TOF MS as a readily available typing method to recognize hospital outbreaks more rapidly than genome-based methods such as PFGE or WGS. While the current “gold-standard” PFGE or WGS require several days or perhaps longer for rare pathogens, MALDI-TOF MS-based typing can be achieved within a single day. This method may prove to be extremely valuable in ongoing outbreak situations with gram-negative pathogens: elucidating quickly whether newly identified isolates belong to the outbreak cluster or not could greatly help to resolve an outbreak.

## Conclusion

In conclusion, we report recurrent clusters of *Agrobacterium* spp*.* bacteremia likely acquired during CT contrast medium injection in two different CT rooms. Also, we highlight the use of MALDI-TOF MS-based typing as a method for rapid identification of outbreak clusters due to rare pathogens.

## Additional file

**Additional file 1: Table S1.** Overview of environmental sampling. Sampling was performed with premoistened (0,9% NaCl) sterile swabs, direct plating of contrast agents (200 μl per sample) and direct wiping of plant material on plates. Plates were incubated at 35 °C in CO2 enriched atmosphere for 6 days. MALDI-TOF MS was used for bacterial identification. **Table S2.** Overview of eight isolates described in this study. The genome datasets are available in the European Nucleotide Archive repository, under Project number PRJEB34002 [https://www.ebi.ac.uk/ena/data/view/PRJEB34002]. **Table S3.** Peak list generated from visual examination of MALDI-TOF spectra of the Isolates A-H. Presence (1) or absence (0) of peaks is indicated at the m/z positions. Potentially double-ionized peaks are indicated with an asterisk (*); numbers in bold indicate peaks with a signal-to-noise ratio > 10. **Table S4.** Results of digital DNA:DNA hybridization for isolates F, H, G and B performed against all known Agrobacterium genomospecies. A dDDH value over 70 (http://ggdc.dsmz.de/; Formula 2) or ANI value over 95% (http://enve-omics.ce.gatech.edu/ani/) indicates the same species. **Table S5.** Comparison of MALDI-TOF MS spectra with a database generated on the base of putatively ascribed ribosomal protein masses extracted from genomic data. G = genomospecies. **Figure S1.** Timeline of clinical, microbiological, environmental, and treatment features of the two Index cases (Cluster I). **Figure S2.** Representative differentiating mass to charge (m/z) peaks. The m/z peak 7882 (*) is present in isolates C (pink), D (violet), E (light green), F (dark green) and H (orange) – the peak at 7924 m/z (**) is present in isolates A (light blue) and B (dark blue). Both peaks are absent in isolate G (red). Four spectra were recorded for each isolate A-H. **Figure S3.** (A) Disposable contrast reservoir with the rubber seal (#) in resting position. The potential contaminable zone lies behind the rubber seal (★). (B) The contrast reservoir is filled with contrast media by pushing (1) the rubber-sealed plunger (*) and pulling it (2) back to resting position. (C) The reservoir is inserted in the injector device system and ready to use. **Figure S4.** Model highlighting the potentially contaminable area of the reservoir behind the rubber seal (★), for visualization a fluorescent solution was used (A: resting position, B: rubber seal pushed in, C: rubber seal back in resting position). **Figure S5.** Principal component analysis: Four spectra were recorded for each isolate A-H. Two-dimensional plot of the first two principal components indicating the two clusters I (isolates A and B) and II (isolates C, D, E and F) and the “unrelated” isolates G and H. **Figure S6.** PFGE based typing of isolates A-H. The scale indicates the degree of similarity (%) as calculated by dice correlation analysis.

## Data Availability

All data generated or analysed during this study are included in this article, its supplementary information files or are available from the corresponding author on reasonable request.
