# Supplementary material for: Agrobacterium spp. nosocomial outbreak assessment using rapid MALDI-TOF MS based typing, confirmed by whole genome sequencing
Source: Antimicrob Resist Infect Control. 2019 Nov 4;8:171. doi: 10.1186/s13756-019-0619-y (PMC6829841; doi:10.1186/s13756-019-0619-y)

Supplementary material

Additional file 1: Table S1

Overview of environmental sampling. Sampling was performed with premoistened (0,9% NaCl) sterile swabs, direct plating of contrast agents (200µl per sample) and direct wiping of plant material on plates. Plates were incubated at 35°C in CO_2 enriched atmosphere for 6 days. MALDI-TOF MS was used for bacterial identification.

Swab site		Date	Detection of *Agrobacterium* spp.
**1. Cardiac catheter laboratory:**			
Contrast media injector	Plunger (head)	2016-02-05	Not detected
	Plunger (body)	2016-02-05	Not detected
	Plunger lodge	2016-02-05	Not detected
	Glass Cylinder	2016-02-05	Not detected
	Disposable contrast media Cylinder	2016-02-05	Not detected
Contrast media solution	Bottle I	2016-02-05	Not detected
	Bottle II	2016-02-05	Not detected
**2. CT Scan room :**			
Contrast media Injector			
	Connecting site to patient tubing set (membrane)	2017-03-03	Not detected
	Connecting site to patient tubing set (lodge)	2017-03-20	Not detected
	Pump lodge	2017-03-20	Not detected
	Tubing set canal I	2017-03-20	Not detected
	Tubing set canal II	2017-03-20	Not detected
	Tubing set canal III	2017-03-20	
Contrast media solution	Contrast media bottle (mebrane)	2017-03-03	Not detected
	Contrast media solution (after flush through injector)	2017-03-03	Not detected
	Contrast media solution (after flush through injector)	2017-03-20	Not detected
	Contrast media solution (after patient)	2017-03-20	Not detected
	Contrast media solution (disposal canister)	2017-03-03	Not detected
	Contrast media solution (bottle)	2017-03-20	Not detected
	Disposal canister (edge)	2017-03-03	Not detected
**3. Radiology Corridor**	Plant I (leaf)	2017-03-03	Not detected
	Plant II leaf)	2017-03-03	Not detected
	Plant III (leaf)	2017-03-03	Not detected

Additional file 1: Table S2

Overview of eight isolates described in this study. The genome datasets are available in the European Nucleotide Archive repository, under Project number PRJEB34002 [https://www.ebi.ac.uk/ena/data/view/PRJEB34002].

Isolate	Strain name	Origin	Date of sampling	Date of isolation	Place of isolation	Species	Read accession number	Outbreak Cluster (MALDI-TOF, WGS, PFGE)
A	AGRBE01	Blood culture	29.11.15	01.12.15	Bern, Switzerland	*Agrobacterium radiobacter* (= A. genomovar G4)	ERX3503501	I
B	AGRBE02	Blood culture	07.12.15	08.12.15	Bern, Switzerland	*Agrobacterium radiobacter* (= A. genomovar G4)	ERX3503502	I
C	AGRBE03	Blood culture	01.10.16	04.10.16	Bern, Switzerland	*Agrobacterium* sp. genomovar G3	ERX3503495	II
D	AGRBE04	Blood culture	12.10.16	15.10.16	Bern, Switzerland	*Agrobacterium* sp. genomovar G3	ERX3503496	II
E	AGRBE05	Blood culture	23.01.17	25.01.17	Bern, Switzerland	*Agrobacterium* sp. genomovar G3	ERX3503497	II
F	AGRBE06	Blood culture	24.02.17	27.02.17	Bern, Switzerland	*Agrobacterium* sp. genomovar G3	ERX3503498	II
G	AGRBE07	Blood culture	23.08.11	27.08.11	Bern, Switzerland	*Agrobacterium pusense* (= A. genomovar G2)	ERX3503500	"unrelated"
H	AGRBE08	Blood culture	02.05.13	04.05.13	Bern, Switzerland	*Agrobacterium* sp. genomovar G3	ERX3503499	"unrelated"

Additional file 1: Table S3

Peak list generated from visual examination of MALDI-TOF spectra of the Isolates A-H. Presence (1) or absence (0) of peaks is indicated at the *m/z* positions. Potentially double-ionized peaks are indicated with an asterisk (*); numbers in bold indicate peaks with a signal-to-noise ratio >10.

Additional file 1: Table S3

m/z		5676						**6121**			**6480**	**6535**					7882	7924
**Isolate**	2796	*2835	2841	2970	2974	**2978**	2994	***3058**	3168	3173	***3238**	***3265**	3293	3316	3357	3645	*3941	*3961
**A**	0	0	0	0	1	0	0	0	0	1	0	1	0	1	0	1	0	1
**B**	0	0	0	0	1	0	0	0	0	1	0	1	0	1	0	1	0	1
**C**	0	1	0	0	0	0	1	0	0	0	1	0	1	0	0	0	1	0
**D**	0	1	0	0	0	0	1	0	0	0	1	0	1	0	0	0	1	0
**E**	0	1	0	0	0	0	1	0	0	0	1	0	1	0	0	0	1	0
**F**	0	1	0	0	0	0	1	0	0	0	1	0	1	0	0	0	1	0
**G**	1	0	1	1	0	1	0	0	1	0	0	1	0	0	0	0	0	0
**H**	0	1	0	0	0	0	1	1	0	0	1	0	1	0	1	0	1	0

m/z		8195	**8232**		8502	**8787**	8797	**8848**	8927	**8984**	**9140**		**9880**	**9953**	**9968**	10076	10510	10525
**Isolate**	3986	*4097	***4115**	4133	*4250	***4393**	*4398	***4425**	*4464	***4493**	***4570**	**4669**	***4941**	***4977**	***4983**	*5039	*5256	*5263
**A**	0	0	0	0	0	0	0	0	1	0	1	1	0	1	0	0	0	0
**B**	0	0	0	0	0	0	0	0	1	0	1	1	0	1	0	0	0	0
**C**	0	0	0	0	0	0	0	0	0	1	1	1	1	0	0	0	0	1
**D**	0	0	0	0	0	0	0	0	0	1	1	1	1	0	0	0	0	1
**E**	0	0	0	0	0	0	0	0	0	1	1	1	1	0	0	0	0	1
**F**	0	0	0	0	0	0	0	0	0	1	1	1	1	0	0	0	0	1
**G**	1	0	1	1	1	0	1	0	1	0	0	0	0	0	1	0	1	0
**H**	0	1	0	0	0	1	0	1	0	1	1	1	1	0	0	1	0	1

m/z									
**Isolate**	5402	5440	5468	5596	**5627**	**6193**	**6218**	8034	**8096**
**A**	1	0	1	1	1	0	1	0	0
**B**	1	0	1	1	1	0	1	0	0
**C**	0	1	0	0	0	1	0	1	1
**D**	0	1	0	0	0	1	0	1	1
**E**	0	1	0	0	0	1	0	1	1
**F**	0	1	0	0	0	1	0	1	1
**G**	1	0	0	1	0	0	0	0	0
**H**	0	1	0	0	0	0	0	1	0

Additional file 1: Table S4

Results of digital DNA:DNA hybridization for isolates F, H, G and B performed against all known *Agrobacterium* genomospecies. A dDDH value over 70 (http://ggdc.dsmz.de/; Formula 2) or ANI value over 95% (http://enve-omics.ce.gatech.edu/ani/) indicates the same species.

Query genome	Comparator strain	Reference genome	dDDH	Model C.I.	Prob. DDH >= 70%	%G+C difference	ANI (Two way)
**F**	***Agrobacterium* genomosp. 3 str. CFBP 6623**	**FBWK01000001-FBWK01000073**	**97.4**	**[96.4 - 98.2%]**	**97.73**	**0.02**	**99.74%**
F	*Agrobacterium* genomosp. 7 str. NCPPB 1641	FCNP01000001-FCNP01000051	37.1	[34.7 - 39.6%]	1.25	0.36	
F	*Agrobacterium* genomosp. 7 str. Zutra 3/1	FBWG01000001-FBWG01000050	37	[34.5 - 39.5%]	1.21	0.67	
F	*A. deltaense* YIC4121	MRDI01000001-MRDI01000027	36.4	[33.9 - 38.9%]	1	0.63	
F	*Agrobacterium* genomosp. 5 str. CFBP 6626	FBWE01000001-FBWE01000038	36.3	[33.8 - 38.8%]	0.98	0.26	
F	*Agrobacterium* genomosp. 13 str. CFBP 6927	FBWH01000001-FBWH01000048	36.2	[33.8 - 38.7%]	0.95	0.18	
F	*A. radiobacter* LMG 140T	MRDG01000001-MRDG01000022	35.4	[33 - 37.9%]	0.75	0.17	
F	*Agrobacterium* genomosp. 6 str. NCPPB 925	FBWM01000001-FBWM01000053	34.7	[32.3 - 37.3%]	0.61	0.12	
F	*„A. fabrum“* = genomosp 8 str C58	AE007869-AE007872	33.8	[31.4 - 36.3%]	0.44	0.13	
F	*Agrobacterium* genomosp. 9 Hayward 0363	FCNQ01000001-FCNQ01000029	34.2	[31.8 - 36.7%]	0.5	0.25	
F	*A. arsenijevicii* KFB330	JWIT01000001-JWIT01000074	33.8	[31.4 - 36.3%]	0.44	0.48	
F	*A. nepotum* 39/7	JWJH01000001-JWJH01000079	33.6	[31.2 - 36.1%]	0.41	0.05	
F	*Agrobacterium* genomosp. 1 str. TT111	FBWA01000001-FBWA01000024	32.3	[29.9 - 34.8%]	0.26	0.71	
F	*A. tumefaciens* P4	CM002258-CM002260	32.3	[29.9 - 34.9%]	0.26	0.69	
F	*A. tumefaciens* Ach5	CP011246-CP011249	32.3	[29.9 - 34.8%]	0.26	0.7	
F	*Agrobacterium* genomosp. 2 str. CFBP 5494	FBVY01000001-FBVY01000049	32.2	[29.7 - 34.7%]	0.25	0.07	
F	*A. pusense* NRCPB10	MRDJ01000001-MRDJ01000029	31.6	[29.2 - 34.1%]	0.2	0.07	
F	*A. larrymoorei* ATCC51759	JADW01000001-JADW01000075	21.9	[19.7 - 24.4%]	0	1.97	
F	*A. rubi* NBRC13261	BBJU01000001-BBJU01000056	21.2	[18.9 - 23.6%]	0	1.88	
F	*A. albertimagni* AOL15	ALJF01000001-ALJF01000055	20.3	[18 - 22.7%]	0	2.06	
**H**	**F**		**97.4**	**[96.4 - 98.2%]**	**97.73**	**0.08**	**99.75%**
**H**	***Agrobacterium* genomosp. 3 str. CFBP 6623**	**FBWK01000001-FBWK01000073**	**97.9**	**[97 - 98.6%]**	**97.84**	**0.06**	**99.76%**
**G**	***Agrobacterium* genomosp. 2 str. CFBP 5494**	**FBVY01000001-FBVY01000049**	**85.3**	**[82.6 - 87.6%]**	**93.88**	**0.01**	**98.09%**
**G**	***A. pusense NRCPB10***	**MRDJ01000001-MRDJ01000029**	**84.9**	**[82.2 - 87.3%]**	**93.68**	**0.02**	**98.31%**
G	*A. deltaense* YIC4121	MRDI01000001-MRDI01000027	35.2	[32.8 - 37.7%]	0.7	0.54	
G	*Agrobacterium* genomosp. 7 str. NCPPB 1641	FCNP01000001-FCNP01000051	35.2	[32.7 - 37.7%]	0.69	0.28	
G	*Agrobacterium* genomosp. 7 str. Zutra 3/1	FBWG01000001-FBWG01000050	35.1	[32.7 - 37.6%]	0.68	0.59	
G	*A. radiobacter* LMG 140T	MRDG01000001-MRDG01000022	34.2	[31.7 - 36.7%]	0.5	0.09	

G	*Agrobacterium* genomosp. 9 Hayward 0363	FCNQ01000001-FCNQ01000029	33.8	[31.4 - 36.3%]	0.45	0.17	
G	*Agrobacterium* genomosp. 6 str. NCPPB 925	FBWM01000001-FBWM01000053	32.7	[30.3 - 35.2%]	0.3	0.21	
G	*„A. fabrum“* = genomosp 8 str C58	AE007869-AE007872	31.9	[29.5 – 34.4%]	0.22	0.22	
G	*Agrobacterium* genomosp. 3 str. CFBP 6623	FBWK01000001-FBWK01000073	32.1	[29.7 - 34.6%]	0.24	0.06	
G	*Agrobacterium* genomosp. 13 str. CFBP 6927	FBWH01000001-FBWH01000048	31.5	[29.1 - 34%]	0.19	0.09	
G	*Agrobacterium* genomosp. 5 str. CFBP 6626	FBWE01000001-FBWE01000038	31.1	[28.7 - 33.6%]	0.16	0.17	
G	*A. arsenijevicii* KFB330	JWIT01000001-JWIT01000074	29.8	[27.4 - 32.3%]	0.1	0.56	
G	*A. nepotum* 39/7	JWJH01000001-JWJH01000079	29.6	[27.2 - 32.1%]	0.09	0.13	
G	*Agrobacterium* genomosp. 1 str. TT111	FBWA01000001-FBWA01000024	28.9	[26.5 - 31.4%]	0.07	0.79	
G	*A. larrymoorei* ATCC51759	JADW01000001-JADW01000075	22	[19.7 - 24.4%]	0	2.06	
G	*A. rubi* NBRC13261	BBJU01000001-BBJU01000056	21.4	[19.1 - 23.8%]	0	1.97	
G	*A. albertimagni* AOL15	ALJF01000001-ALJF01000055	21.1	[18.9 - 23.5%]	0	1.97	
**B**	***A. radiobacter* LMG 140T**	**MRDG01000001-MRDG01000022**	**83.9**	**[81.2 - 86.4%]**	**93.17**	**0.29**	**98.18%**
B	*Agrobacterium* genomosp. 7 str. NCPPB 1641	FCNP01000001-FCNP01000051	45.4	[42.9 - 48%]	8.88	0.1	
B	*Agrobacterium* genomosp. 7 str. Zutra 3/1	FBWG01000001-FBWG01000050	45.2	[42.6 - 47.8%]	8.51	0.21	
B	*A. deltaense* YIC4121	MRDI01000001-MRDI01000027	44.5	[41.9 - 47%]	7.38	0.17	
B	*Agrobacterium* genomosp. 9 Hayward 0363	FCNQ01000001-FCNQ01000029	41.2	[38.7 - 43.8%]	3.66	0.21	
B	*Agrobacterium* genomosp. 6 str. NCPPB 925	FBWM01000001-FBWM01000053	37.2	[34.7 - 39.7%]	1.27	0.59	
B	*„A. fabrum“* = genomosp 8 str C58	AE007869-AE007872	36.1	[33.6 – 38.6%]	1.17	0.26	
B	*Agrobacterium* genomosp. 13 str. CFBP 6927	FBWH01000001-FBWH01000048	36.1	[33.6 - 38.6%]	0.92	0.59	
B	*Agrobacterium* genomosp. 3 str. CFBP 6623	FBWK01000001-FBWK01000073	35.5	[33 - 38%]	0.77	0.44	
B	*Agrobacterium* genomosp. 5 str. CFBP 6626	FBWE01000001-FBWE01000038	35	[32.5 - 37.5%]	0.65	0.2	
B	*A. pusense* NRCPB10	MRDJ01000001-MRDJ01000029	34.4	[32 - 36.9%]	0.54	0.39	
B	*Agrobacterium* genomosp. 2 str. CFBP 5494	FBVY01000001-FBVY01000049	34.3	[31.9 - 36.9%]	0.53	0.39	
B	*A. arsenijevicii* KFB330	JWIT01000001-JWIT01000074	34.2	[31.8 - 36.7%]	0.51	0.94	
B	*A. nepotum* 39/7	JWJH01000001-JWJH01000079	33.9	[31.5 - 36.4%]	0.46	0.51	
B	*Agrobacterium genomosp. 1 str. TT111*	FBWA01000001-FBWA01000024	32.7	[30.3 - 35.2%]	0.3	1.17	
B	*A. larrymoorei* ATCC51759	JADW01000001-JADW01000075	22.2	[20 - 24.7%]	0	2.43	
B	*A. rubi* NBRC13261	BBJU01000001-BBJU01000056	21.4	[19.2 - 23.8%]	0	2.34	
B	*A. albertimagni* AOL15	ALJF01000001-ALJF01000055	20.4	[18.2 - 22.8%]	0	1.59	

Additional file 1: Table S5

Comparison of MALDI-TOF MS spectra with a database generated on the base of putatively ascribed ribosomal protein masses extracted from genomic data. G= genomospecies.

Isolate	Identification with SSp 1)	% ID	Identification with Compare/PAPMID™ 2)	Assigned genomospecies (genome)
A	*Agrobacterium radiobacter* G4	99.9	*Agrobacterium radiobacter* G4	***Agrobacterium radiobacter* G4** (Genome B14095)
	Agrobacterium radiobacter G4	99.9	*Agrobacterium radiobacter* G4	
B	*Agrobacterium radiobacter* G4	99.9	*Agrobacterium radiobacter* G4	***Agrobacterium radiobacter* G4** (Genome B14095)
	Agrobacterium radiobacter G4	99.9	*Agrobacterium radiobacter* G4	
C	*Agrobacterium* G3	99.9	*Agrobacterium* G3	***Agrobacterium* G3** (Genome LC34)
	Agrobacterium G3	99.9	*Agrobacterium* G3	
D	*Agrobacterium* G3	99.9	*Agrobacterium* G3	***Agrobacterium* G3** (Genome LC34)
	Agrobacterium G3	99.9	*Agrobacterium* G3	
E	*Agrobacterium* G3	99.9	*Agrobacterium* G3	***Agrobacterium* G3** (Genome LC34)
	Agrobacterium G3	99.9	*Agrobacterium* G3	
F	*Agrobacterium* G3	99.9	*Agrobacterium* G3	***Agrobacterium* G3** (Genome LC34)
	Agrobacterium G3	99.9	*Agrobacterium* G3	
G	*Agrobacterium pusense* G2	99.9	*Agrobacterium pusense* G2	***Agrobacterium pusense* G2** (Genome S33)
	Agrobacterium pusense G2	99.9	*Agrobacterium pusense* G2	
H	*Agrobacterium* G3	99.9	*Agrobacterium* G3	***Agrobacterium* G3** (Genome LC34)
	Agrobacterium G3	99.9	*Agrobacterium* G3	

1) based on validated biomarker mass sets integrated in the SERAMIS database (AnagnosTec, Germany)

2) Putative Assigned Protein Masses for Identification Database (PAPMID™) (Mabritec AG, Switzerland)

Additional file 1: Figure S1

Timeline of clinical, microbiological, environmental, and treatment features of the two Index cases (Cluster I)

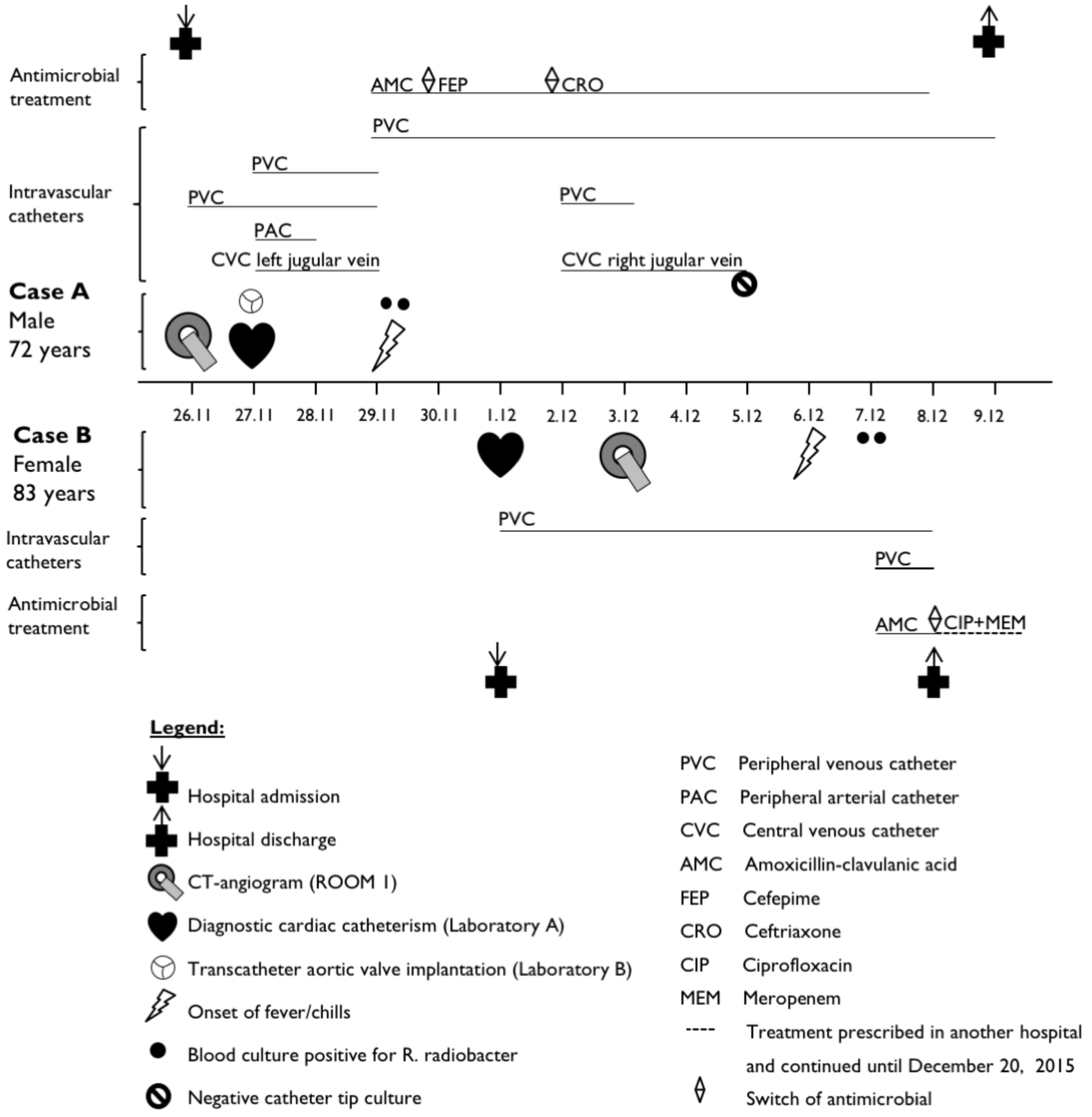

Additional file 1: Figure S2

Representative differentiating mass to charge *(m/z)* peaks. The *m/z* peak 7882 (*) is present in isolates C (pink), D (violet), E (light green), F (dark green) and H (orange) – the peak at 7924 *m/z* (**) is present in isolates A (light blue) and B (dark blue). Both peaks are absent in isolate G (red). Four spectra were recorded for each isolate A-H.

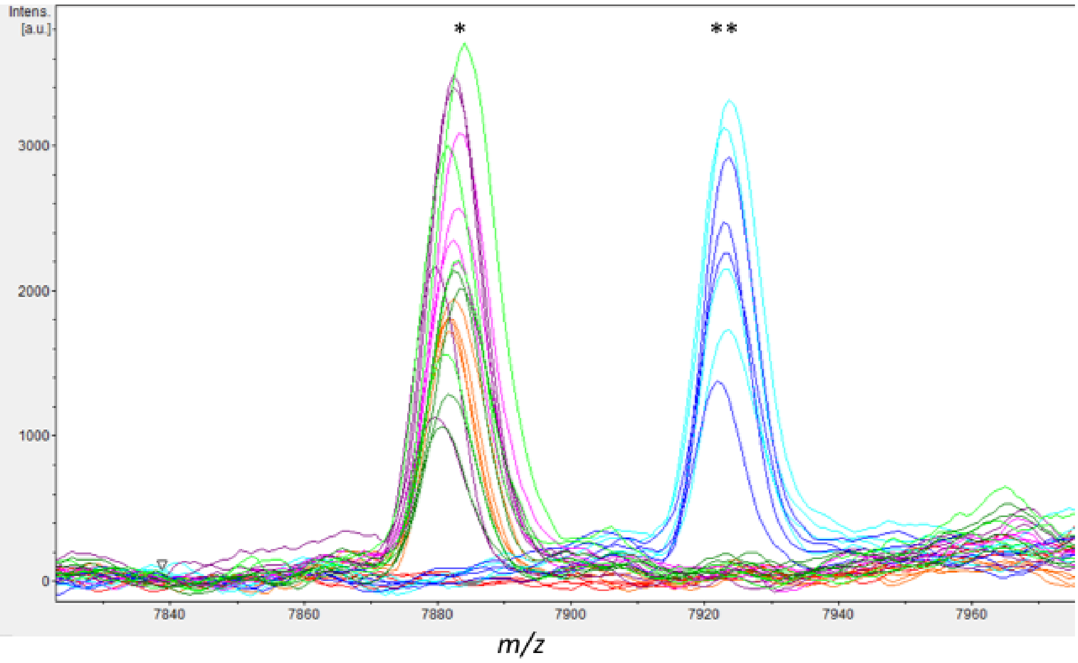

Additional file 1: Figure S3

(A) Disposable contrast reservoir with the rubber seal (#) in resting position. The potential contaminable zone lies behind the rubber seal (★). (B) The contrast reservoir is filled with contrast media by pushing (1) the rubber-sealed plunger (*) and pulling it (2) back to resting position. (C) The reservoir is inserted in the injector device system and ready to use.

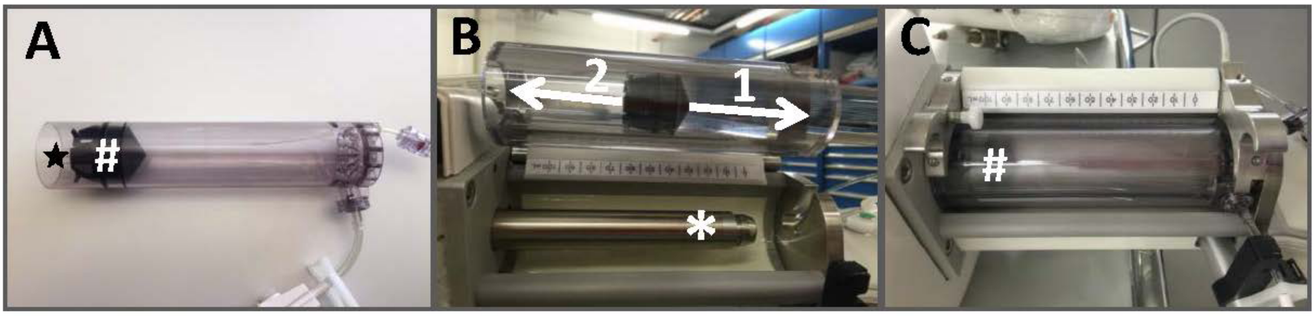

Additional file 1: Figure S4

Model highlighting the potentially contaminable area of the reservoir behind the rubber seal (★), for visualization a fluorescent solution was used (A: resting position, B: rubber seal pushed in, C: rubber seal back in resting position).

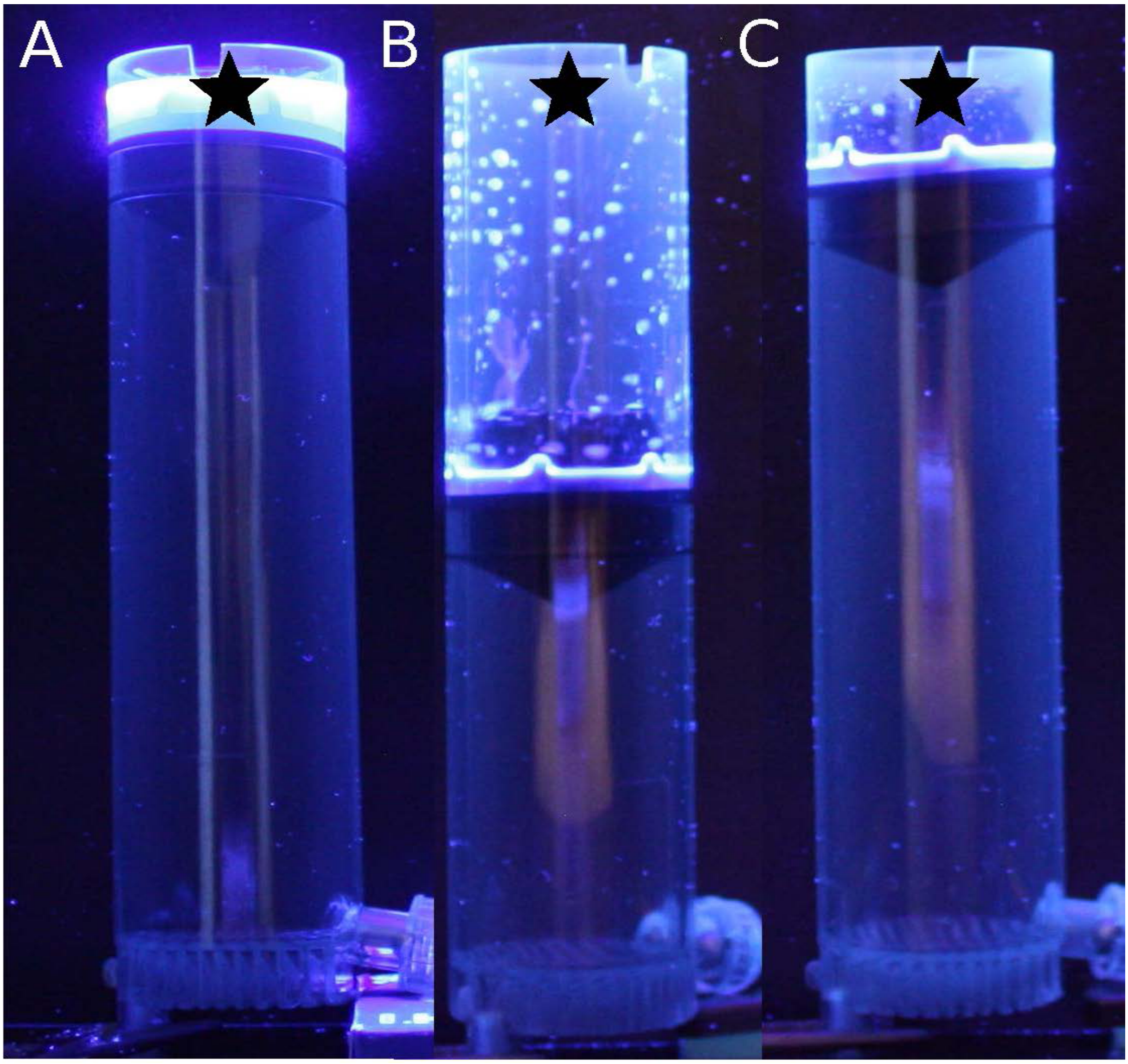

Additional file 1: Figure S5

Principal component analysis: Four spectra were recorded for each isolate A-H. Two-dimensional plot of the first two principal components indicating the two clusters I (isolates A and B) and II (isolates C, D, E and F) and the “unrelated” isolates G and H.

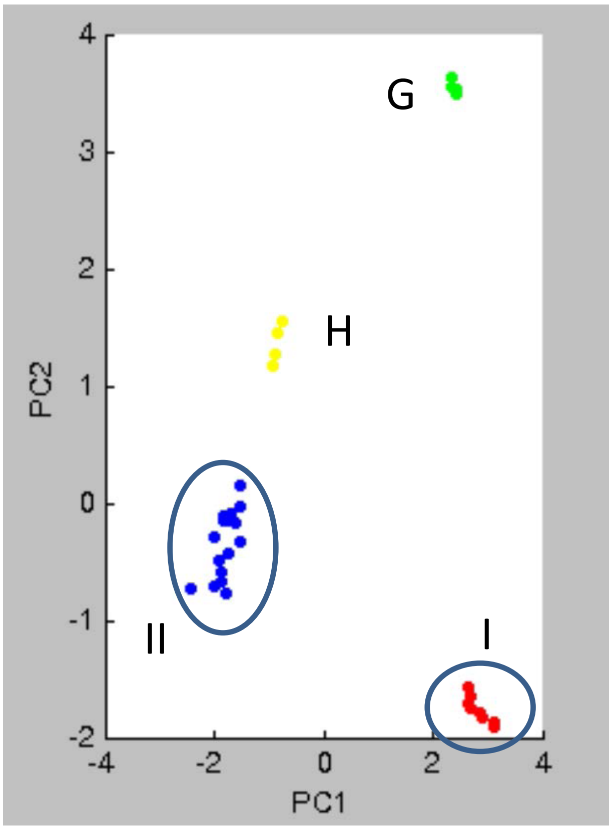

Additional file 1: Figure S6

PFGE based typing of isolates A-H. The scale indicates the degree of similarity (%) as calculated by dice correlation analysis.

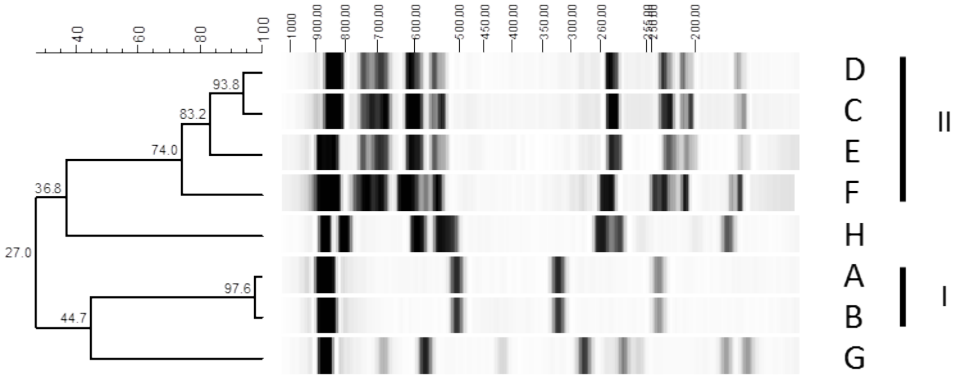

Supplement: Supplementary file 1 — Additional file 1: Table S1. Overview of environmental sampling. Sampling was performed with premoistened (0,9% NaCl) sterile swabs, direct plating of contrast agents (200 μl per sample) and direct wiping of plant material on plates. Plates were incubated at 35 °C in CO2 enriched atmosphere for 6 days. MALDI-TOF MS was used for bacterial identification. Table S2. Overview of eight isolates described in this study. The genome datasets are available in the European Nucleotide Archive repository, under Project number PRJEB34002 [https://www.ebi.ac.uk/ena/data/view/PRJEB34002]. Table S3. Peak list generated from visual examination of MALDI-TOF spectra of the Isolates A-H. Presence (1) or absence (0) of peaks is indicated at the m/z positions. Potentially double-ionized peaks are indicated with an asterisk (*); numbers in bold indicate peaks with a signal-to-noise ratio > 10. Table S4. Results of digital DNA:DNA hybridization for isolates F, H, G and B performed against all known Agrobacterium genomospecies. A dDDH value over 70 (http://ggdc.dsmz.de/; Formula 2) or ANI value over 95% (http://enve-omics.ce.gatech.edu/ani/) indicates the same species. Table S5. Comparison of MALDI-TOF MS spectra with a database generated on the base of putatively ascribed ribosomal protein masses extracted from genomic data. G = genomospecies. Figure S1. Timeline of clinical, microbiological, environmental, and treatment features of the two Index cases (Cluster I). Figure S2. Representative differentiating mass to charge (m/z) peaks. The m/z peak 7882 (*) is present in isolates C (pink), D (violet), E (light green), F (dark green) and H (orange) – the peak at 7924 m/z (**) is present in isolates A (light blue) and B (dark blue). Both peaks are absent in isolate G (red). Four spectra were recorded for each isolate A-H. Figure S3. (A) Disposable contrast reservoir with the rubber seal (#) in resting position. The potential contaminable zone lies behind the rubber seal (★). (B) The contrast r [file 13756_2019_619_MOESM1_ESM.pdf]
